# Neurophysiological visual assessment in patients with idiopathic intracranial hypertension: visual evoked potential and multifocal field electroretinography

**DOI:** 10.1186/s12883-023-03220-8

**Published:** 2023-05-10

**Authors:** Marwa A. Elgaly, Hanan Hosny, Hala R. El Habashy, Mona Hussein, Rehab Magdy, Rehab Elanwar

**Affiliations:** 1 Neuro Diagnostic Research Center (NDRC), Beni-Suef University, Beni-Suef, Egypt; 2 Neuro Diagnostic Research Center (NDRC), Beni-Suef University, Cairo, Egypt; 3 Department of Clinical Neurophysiology, Cairo University, Cairo, Egypt; 4 Department of Neurology, Beni-Suef University, Beni-Suef, Egypt; 5 Department of Neurology, Cairo University, Cairo, Egypt; 6 Neuro Diagnostic Research Center (NDRC), Beni-Suef University, Salah Salem Street, Beni-Suef, 62511 Egypt

**Keywords:** Idiopathic intracranial hypertension, VEP, mf-ERG, CSF pressure, Papilledema

## Abstract

**Background:**

Determining the cause of visual deterioration in idiopathic intracranial hypertension (IIH) patients is of clinical necessity. This study aimed to study the effect of chronic increased ICP on the retina and optic nerve through objective electrophysiological measures in chronic IIH patients.

**Methods:**

Thirty patients with chronic IIH and thirty age and sex-matched healthy controls were included in this study. Papilledema grade and CSF pressure were evaluated in the patients’ group. Both groups were submitted to visual evoked potentials (VEP) and multifocal electroretinogram (mfERG).

**Result:**

The mean value of P100 latencies of the right and left on two check sizes, 1 deg and 15ṁ in chronic IIH patients, was significantly delayed than controls (P-value < 0.001 for each). Chronic IIH patients showed a significantly lower amplitude of the right and left R1, R2, R3, R4 & R5 compared to controls (P-value < 0.001, < 0.001) (P-value < 0.001, < 0.001) (P-value < 0.001, < 0.001) (P-value < 0.001, = 0.001) (P-value = 0.002, < 0.001), respectively. Also, patients showed a significantly delayed peak time of the right and left R1 and R2 compared to controls (P-value < 0.001, < 0.001) (P-value = 0.001, = 0.009), respectively. There was a significant positive correlation between each of CSF pressure and papilledema grade with right and left PVEP latencies. In contrast, there was no statistically significant correlation between either CSF pressure or papilledema grade and PVEP amplitudes in both eyes.

**Conclusion:**

In chronic IIH patients, both optic nerve dysfunction and central retinal changes were identified, supported by VEP and the mfERG findings.

## Introduction

Idiopathic intracranial hypertension (IIH) is a condition of increased intracranial pressure (ICP) of unknown etiology, which can eventually cause papilledema and visual deterioration [1]. Vision loss is often reversible if treatment is started in time but can be permanent in up to 40% of patients.

In addition to papilledema and optic nerve atrophy, several retinal changes may contribute to the visual deficit in IIH, including choroidal compression across the macula, choroidal neovascularization, and axoplasmic stasis in the retinal ganglion cells (RGCs) due to compression by the elevated ICP which lead to RGCs dysfunction [2, 3].

Currently, perimetry is the usual method for evaluating the visual system in patients with IIH [4]. Nevertheless, electrophysiological measures of the visual system are of imperative importance in research settings because they are rapid, objective, non-invasive, and require minimal patient attention [5].

The Visual evoked potential (VEP) measures the integrated function of the optic nerve and post-retinal visual pathway dysfunction to the occipital lobe [6]. Some studies demonstrated VEPs abnormalities even in patients without apparent central nervous system involvement [7, 8]. Abnormal prolonged P100 latency was reported in some IIH patients [9, 10].

Multifocal electroretinography (mfERG) is another reliable method that simultaneously stimulates multiple retinal regions (central to mid-peripheral) and records each electrophysiological response independently. It can detect small retinal dysfunction in central to mid-peripheral regions [11].

This work aimed to clarify the effect of chronic increased ICP on the retina and optic nerve by studying the multifocal electroretinography (mfERG) findings and visual evoked potential (VEPs) in patients with chronic IIH.

## Methods

### Study design and participants

This case-control study was conducted from the period of 1/1/2019 to 30/12/2020. Thirty female patients over 18 years of age, diagnosed with chronic IIH for at least six months, were recruited from the Neurology clinic at Beni-Suef University Hospital. Diagnosis of IIH was established according to the modified Dandy criteria [12]. Another thirty age-matched healthy females were recruited from health care workers as a control group.

Exclusion criteria for the two groups included: ocular diseases (e.g., uveitis, cataract, or glaucoma), concurrent other neurological, autoimmune, inflammatory, or medical illnesses known to affect the visual system (e.g., diabetic retinopathy), history of exposure to drugs such as minocycline, cyclosporine, hydroxychloroquine, topiramate, ethambutol, and anticholinergics, substances known to affect vision such as lead, heavy metals, ethylene glycol, alcohol, and industrial agents. Pregnant patients were also excluded.

### Clinical assessment

The patients and control groups were subjected to detailed neuro-ophthalmological assessment in the Ophthalmology clinic at Beni-Suef University Hospital, including visual acuity testing with a Snellen chart and ophthalmoscopic examination for determining papilledema grade.

Lumbar puncture was performed for patients to measure CSF pressure using a standard 18- or 20-gauge spinal needle and a manometer positioned at a 90-degree angle to the spine. The opening pressure was measured while the subject was placed in a lateral decubitus position.

### Neurophysiological assessment

All neurophysiological studies were performed in the Neurodiagnostic and Research Center (NDRC), Beni Suef University Hospital utilizing Reti-Scan 21 (Roland Consult, Brandenburg a.d. Havel, Germany) Roland RETI system (Roland, Germany) including:

#### 1- Visual evoked potential (VEP)

T.V pattern reversing checkerboard of black and white checks was recorded from each eaye separately using 1 degree and 15 min of arc at contrast according to the International Society for Clinical Electrophysiology of Vision (ISCEV) standards for visual evoked potentials (2011) [13].

The EEG sliver cup active electrode was placed on the midline occipital area (Oz), reference electrode on mid frontal (Fz), and ground electrode over the mastoid according to standardized ‘‘International 10/20system’’ keeping the impedance below 5 K Ohm.

VEP components are termed N75, P100, and N145 regarding their polarity. The P100 responses regarding latency (in msec.) and amplitudes in millivolts (mv) were assessed. The amplitude of P100 response was measured from the peak N75 to the trough of P100. The most consistent waveform of the pattern-reversal VEP is the P100, generated and recorded over the occipital lobe [14].

#### 2- Multifocal Electroretinogram (mfERG)

The electrical responses from the retina were recorded monocularly (right and left eyes) with active HK loop electrode, reference, and ground silver EEG electrodes. The HK loop electrode was located at the inferior eyelid and its loop contacting the globe just below the cornea, the reference electrode was on the ipsilateral ear, and the ground electrode was sited on the forehead maintaing the impedance below 5 K Ohm, the gain was 200µV and bandpass filter range was from 10 HZ to 100 HZ according to International Society for Clinical Electrophysiology in Vision (ICSEV) standards for clinical multifocal electroretinography (2011 edition) [15].

After cleaning the skin with alcohol and propreb, and after 10 min of light adaptation, anesthetic was dropped. The pupil was dilated for at least 8 mm with tropicamide hydrochloride 1%.

The subject was fixated her eye on a red cross in the center of the motivating screen. The stimulus was produced using a cathode ray tube (CRT) delivery system. It consisted of 61 hexagons, involving 25°–30° of the visual field and exhibting on a 20-inch screen at a insepecting space of 33 cm. The luminance of each hexagon on the screen was 100–200 cd/m2 in the lighted state and < 1 cd/m2 in the dark state. The mean screen luminance during study was 50–100 cd/m2, and the contrast between white and black hexagons should be greater for 90%. The circumtance region of the CRT luminance was equal to the mean luminance of the stimulus array.

Each session took 6 min and was divided into 47-second segments, and eight runs were recorded in total.

The mfERG records focal electrophysiological responses from dissimilar regions of the retina, and the location of the mfERG stimuli and anatomical zone nearly corresponded as follows:

Regarding the mfERG rings: ring 1 to the fovea, ring 2 to the parafovea, ring 3 to the perifovea, ring 4 to the near periphery, and ring 5 to the central part of the middle periphery [16].

Regarding the mfERG quadrants: quadrant 1 to the lower nasal, quadrant 2 to the upper nasal, quadrant 3 to the upper temporal, and quadrant 4 to the lower temporal [16].

For each hexagon, the peak time of the P1 wave and trough to peak amplitude of the P1 wave was calculated. Average responses were calculated for the five retinal rings and the four retinal quadrants [16].

### Sample size

The sample size was calculated using G*Power version 3.1.9.7 Software based on the results of a pilot study we had done before starting our study. The type of power analysis was: A priori: compute required sample size- given α, power, and effect size. The input parameters were: Allocation ratio N2/N1 = 1, effect size = 0.66, α err prob = 0.05, and power (1-β err prob) = 0.80. The output parameters were: noncentrality parameter δ = 2.558, critical t = 1.672, and Df = 58. A total sample size of 30 partipatients in each group was required to achieve a statistical power (1–β) 80%.

### Ethical statement

The study was approved by the Faculty of Medicine, Beni -Suef University research ethical committee (Approval number is FWA00015574). Informed written consent was obtained from participants.

### Statistical analysis

Statistical analysis was performed using SPSS software version 20. Categorical variables such as papilledema grading in BIH patients were presented as numbers and percentages. Quantitative data in BIH patients and controls such as age, CSF pressure, VEP, and mfERG parameters were presented as mean and standard deviation (SD). Chi-square test was used to compare between both eyes in papilledema grading. Independent samples T-test was used to compare between BIH patients and controls in VEP and mfERG parameters. Pearson’s correlation was used to test the association between CSF pressure, papilledema grading and both VEP and mfERG parameters. P-values ≤ 0.05 (2-sided) were considered statistically significant.

## Results

The mean age of the patients was 36.13 ± 10.28, whereas the mean age of the control group was 36.96 ± 7.77 (P-value 0.725). The mean value for CSF pressure of chronic IIH patients was 317.66 ± 63.82 mm H2O. Papilledema grading in the right eyes was as follows: grade I in one patient (3.3%), grade II in 17 (56.7%), and grade III in 12 (40%). For the left eyes, grade I papilledema was reported in 2 (6.7%), grade II in 13 (43.3%), and grade III in 15 (50%). There was no statistically significant difference between both eyes regarding papilledema grading (P-value = 0.549).

The CSF pressure was significantly correlated with the papilledema grade in the RT eye (r coef.= 0.404, P-value = 0.027). But, there was no statistically significant correlation between CSF pressure and the papilledema grade in the LT eye (r coef.= 0.121, P-value = 0.524).

### Neurophysiological assessment of IIH patients and control groups

For both eyes, the mean value of P100 latencies of chronic IIH was significantly delayed than controls on two check sizes, 1 deg and 15ṁ (Table 1). However, no statistically significant difference was found between patients and controls regarding the mean value of P100 amplitudes on the two check sizes (Table 1).

**Table 1:** Values of VEP in patients and control groups

	Patients (*n* = 30)	Controls (*n* = 30)	*P*-value
RT P100 latency 1°	109.16 (11.73)	94.85 (4.40)	< 0.001*
RT P100 amplitude 1°	11.36 (3.86)	12.1 (4.93)	0.522
RT P100 latency 15´	109.88 (10.46)	97.35 (4.23)	< 0.001*
RT P100 amplitude 15´	11.73 (5)	11.95 (3.3)	0.845
LT P100 latency 1°	113.68 (13.2)	96.53 (4.25)	< 0.001*
LT P100 amplitude 1°	11.92 (5.71)	13.7 (5.79)	0.234
LT P100 latency 15´	111.42 (11.5)	97.13 (4.32)	< 0.001*
LT P100 amplitude 15´	10.82 (4)	12.67 (4.54)	0.099

VEP: visual evoked potential, ***P***-value is significant if ˂ 0.05

The mf-ERG data were studied for the mean and SD of the peak time (ms) and amplitude (nv/deg) of the right and left five retinal rings and the four quadrants.

Chronic IIH patients showed a significantly lower amplitude of the right and left R1, R2, R3, R4 & R5 compared to controls (P-value < 0.001, < 0.001) (P-value < 0.001, < 0.001) (P-value < 0.001, < 0.001) (P-value < 0.001, = 0.001) (P-value = 0.002, < 0.001) respectively (Table 2).

**Table 2:** Values of mfERG in five rings in patients and control groups

MfERG Rings	Patients (*n* = 30)	Controls (*n* = 30)	*P*-value
RT R1 amplitude	57.08 (16.48)	102.09 (25.67)	< 0.001*
RT R1 peak time	47.14 (4.28)	42.23 (4.17)	< 0.001*
LT R1 amplitude	52.02 (16.25)	100.48 (23.13)	< 0.001*
LT R1 peak time	46.16 (3.5)	41.59 (4.43)	< 0.001*
RT R2 amplitude	29.27 (9.5)	41.96 (7.33)	< 0.001*
RT R2 peak time	45.35 (3.24)	42.3 (3.71)	0.001*
LT R2 amplitude	26.58 (8.32)	42.27 (8.81)	< 0.001*
LT R2 peak time	45.28 (2.54)	43.25 (3.24)	0.009*
RT R3 amplitude	19.01 (5.11)	26.6 (4.15)	< 0.001*
RT R3 peak time	44.26 (2.58)	43.61 (2.40)	0.315
LT R3 amplitude	18.99(4.47)	25.54 (3.32)	< 0.001*
LT R3 peak time	44.18 (3.36)	42.47 (3.47)	0.058
RT R4 amplitude	12.14 (3.80)	16.34 (4.16)	< 0.001*
RT R4 peak time	43.61 (2.80)	43.18 (2.61)	0.541
LT R4 amplitude	12.25 (3.63)	15.66 (3.69)	0.001*
LT R4 peak time	42.90 (3.86)	42.5 (3.6)	0.685
RT R5 amplitude	10.16 (3.47)	12.77 (2.77)	0.002*
RT R5 peak time	43.22 (2.46)	43.06 (1.93)	0.772
LT R5 amplitude	9.26 (2.62)	12.29 (2.94)	< 0.001*
LT R5 peak time	43.42 (2.16)	42.49 (3.09)	0.185

mfERG: Multifocal electroretinography, ***P***-value is significant if ˂ 0.05

Also, the patients showed a significantly delayed peak time of the right and left R1 and R2 compared to controls (P-value < 0.001, < 0.001) (P-value = 0.001, = 0.009), respectively (Table 2).

Furthermore, chronic IIH patients showed a significantly lower amplitude of the right and left Q1, Q2, Q3 & Q4 compared to controls (P-value < 0.001, < 0.001) (P-value < 0.001, < 0.001) (P-value < 0.001, < 0.001) (P-value < 0.001, = 0.001), respectively (Table 3). In contrast, There was no statistically significant difference in the peak time in four retinal quadrants of the two eyes between chronic IIH patients and controls, respectively (Table 3).

**Table 3:** Values of mfERG in four quadrants in patients and control groups

MfERG Quadrants	Patients (*n* = 30)	Controls (*n* = 30)	*P*-value
RT Q1 amplitude	11.59 (3.53)	18.97 (4.10)	< 0.001*
RT Q1 peak time	44.35 (4.38)	43.08 (2.95)	0.194
LT Q1amplitude	11.86 (4.76)	19.41 (3.34)	< 0.001*
LT Q1peak time	43.70 (2.94)	42.58 (3.04)	0.153
RT Q2 amplitude	13.94 (3.85)	18.42 (3.79)	< 0.001*
RT Q2 peak time	42.88 (3.14)	42.64 (2.95)	0.768
LT Q2 amplitude	12.85 (3.05)	19.55 (4.27)	< 0.001*
LT Q2 peak time	43.32 (3.25)	42.95 (2.19)	0.608
RT Q3 amplitude	13 (3.94)	19.44 (4.21)	< 0.001*
RT Q3 peak time	43.38 (3.29)	42.84 (3.09)	0.516
LT Q3 amplitude	13.08 (4.16)	19.35 (3.26)	< 0.001*
LT Q3 peak time	44.24 (2.62)	43.47 (3.29)	0.323
RT Q4 amplitude	10.31 (3.25)	18.14 (2.80)	< 0.001*
RT Q4 peak time	43.09 (2.96)	42.65 (2.65)	0.541
LT Q4 amplitude	9.95 (3.11)	18.64 (3.09)	< 0.001*
LT Q4 peak time	43.93 (3.42)	43.54 (3.16)	0.649

mfERG: Multifocal electroretinography, ***P***-value is significant if ˂ 0.05

### Neurophysiological measures in relation to clinical data in IIH patients

There was a significant positive correlation between CSF pressure and right and left PVEP latencies (P-value < 0.001, < 0.001, 0.009, 0.008, respectively) (Table 4). However, there was no statistically significant correlation between CSF pressure and PVEP amplitudes in both eyes.

**Table 4:** Correlations between VEP measures and clinical parameters in patients group

PVEP	CSF pressure		Papilledema	
	(*r*) coef.	*P*-value	(*r*) coef.	*P*-value
RT P100 latency 1°	0.649	< 0.001*	0.617	< 0.001*
RT P100 amplitude1°	0.229	0.224	-0.086	0.652
RT P100 latency 15´	0.647	< 0.001*	0.738	< 0.001*
RT P100 amplitude15´	-0.069	0.717	-0.309	0.097
LT P100 latency 1°	0.471	0.009*	0.522	0.002*
LT P100 amplitude 1°	-0.301	0.106	-0.293	0.116
LT P100 latency 15´	0.414	0.008*	0.513	0.004*
LT P100 amplitude 15´	-0.197	0.296	-0.235	0.212

VEP: visual evoked potential, ***P***-value is significant if ˂ 0.05

There was a statistically significant correlation between papilledema grade in both eyes and PVEP latencies (P-value < 0.001, < 0.001, 0.002, 0.004, respectively) (Table 4). However, there was no statistically significant correlation between papilledema grade and PVEP amplitudes in both eyes.

There was no statistically significant correlation between either CSF pressure or papilledema grade and right and left R1, R2, R3, R4 & R5 amplitude and peak time (Table 5). Moreover, There was no statistically significant correlation between either CSF pressure or papilledema grade and right and left Q1, Q2, Q3& Q4 amplitude, and peak time (Table 6).

**Table 5:** Correlations between mfERG measures in the five rings and clinical parameters in patients group

MfERG Rings	CSF pressure		Papilledema	
	(*r*) coef.	*P*-value	(*r*) coef.	*P*-value
RT R1 amplitude	-0.085	0.656	0.095	0.617
RT R1 peak time	-0.123	0.518	0.249	0.798
LT R1 amplitude	-0.185	0.329	-0.053	0.783
LT R1 peak time	-0.007	0.970	-0.185	0.328
RT R2 amplitude	-0.147	0.438	-0.272	0.145
RT R2 peak time	0.384	0. 366	0.150	0.428
LT R2 amplitude	0.118	0.533	-0.115	0.547
LT R2 peak time	0.011	0.955	-0.097	0.610
RT R3 amplitude	-0.186	0.325	-0.141	0.458
RT R3 peak time	-0.144	0.449	-0.342	0.065
LT R3 amplitude	-0.073	0.700	-0.096	0.613
LT R3 peak time	0.165	0.382	-0.154	0.415
RT R4 amplitude	-0.072	0.706	0.086	0.653
RT R4 peak time	0.152	0.424	0.029	0.879
LT R4 amplitude	-0.111	0.560	-0.347	0.060
LT R4 peak time	-0.138	0.465	0.239	0.203
RT R5 amplitude	-0.132	0.488	0.015	0.936
RT R5 peak time	-0.201	0.286	-0.098	0.607
LT R5 amplitude	-0.068	0.721	0.005	0.979
LT R5 peak time	0.086	0.652	0.074	0.699

mfERG: Multifocal electroretinography, ***P***-value is significant if ˂ 0.05

**Table 6:** Correlations between mfERG measures in four quadrants and clinical parameters in patients group

MfERG Quadrants	CSF pressure		Papilledema	
	(*r*) coef.	*P*-value	(*r*) coef.	*P*-value
RT Q1 amplitude	0.063	0.743	0.045	0.815
RT Q1 peak time	0.087	0.648	0.036	0.850
LT Q1 amplitude	-0.044	0.816	0.077	0.686
LT Q1 peak time	0.067	0.725	0.144	0.447
RTQ2 amplitude	0.018	0.927	0.322	0.083
RT Q2peak time	-0.068	0.721	0.148	0.434
LT Q2 amplitude	-0.264	0.159	0.159	0.401
LT Q2 peak time	-0.318	0.086	-0.284	0.129
RT Q3 amplitude	-0.025	0.895	0.044	0.816
RT Q3 peak time	-0.132	0.488	0.135	0.478
LT Q3 amplitude	0.087	0.646	-0.138	0.467
LT Q3 peak time	-0.310	0.096	-0.024	0.898
RT Q4 amplitude	-0.120	0.527	-0.071	0.710
RT Q4 peak time	0.179	0.345	0.109	0.565
LT Q4 amplitude	-0.090	0.638	-0.249	0.115
LT Q4 peak time	-0.238	0.206	0.261	0.164

mfERG: Multifocal electroretinography, ***P***-value is significant if ˂ 0.05

## Discussion

The present study gave insight into objective electrophysiological measures of visual function in IIH patients and their applicability to complement the clinical examination that may potentially improve management decisions.

The current results agreed with [9, 17], who reported abnormally delayed VEP latencies in IIH patients with normal amplitudes, suggesting demyelinating rather than axonal effects on the optic nerves [18]. Furthermore, this study declared that VEP could be used as a quantitative indicator of optic nerve damage secondary to compression in elevated ICP, demonstrated by the significant correlations between VEP latencies and each papilledema grading and CSF pressure. There is considerable evidence that the primary mechanism for damage to the optic nerve in IIH is the disruption of axonal transport. It is likely that high CSF pressure disturbs the normal gradient between intraocular and retrolaminar pressure and results in increased tissue pressure within the optic nerve. Another potential mechanism for damage to the optic nerve in IIH is the intraneuronal optic nerve ischemia due to compression of small arterioles.[19, 20].

Although optic nerve compression is the commonly accepted contributing pathology of visual deterioration in cases of IIH [21], the present study’s findings give an imperative perspective to the outer retinal involvement that might complicate the course of IIIH, evidenced by the mfERG results. Distinguishing outer retinal changes from optic neuropathy as the cause of visual deterioration is decisive because outer retinal changes in the macula could often be reversible [3, 22].

To the best of our knowledge, this is the first study that explored the outer retinal function in IIH patients by mfERG. Outer retinal function was previously evaluated in IIH patients with full-field electroretinography (ffERG) by JC Park, HE Moss and JJ McAnany [23], who found a more significant ffERG amplitude reduction in patients than in control. However, the mfERG used in the current study provides more precise information than ffERG. The former registers the response of multiple areas in the central retina rather than the global retinal response captured by the latter [24].

It is well established that mfERG responses are generated mainly by cons photoreceptors and bipolar retinal cells. Hence, such cell damage may lead to amplitude reduction or prolonged peak time measurements on mfERG responses [24, 25]. In this study, the mfERG amplitudes showed a significant reduction in all rings through all quadrants of both eyes, with delayed peak times only in retinal rings 1 and 2 compared to the controls. These findings may indicate a diffuse dysfunction of the macular cones and bipolar retinal cells, with more affection for the central part of the macula (fovea and parafovea) than the peripheral region.

In a systematic review conducted by P Nichani and JA Micieli [22], several central manifestations were described in the outer retina in IIH patients, including chorioretinal folds, macular exudate, choroidal infraction, and macular edema. All these observations might account for the abnormal retinal responses on the mfERG seen in our IIH patients.

Interestingly, the significantly delayed peak time of the mfERG P1 wave in retinal rings 1 and 2 (foveal and parafoveal) could be attributed to macular edema or exudate in IIH patients. The hard exudates in the macular region were reported to prolong the implicit time assessed with mfERG [26].

Moreover, the present study revealed a non-significant correlation with retinal response in mfERG, in harmony with P Nichani and JA Micieli [22], who found that the retinal manifestation associated with IIH could reduce visual acuity in IIH patients independently of papilledema.

In the present study there was no statistically significant difference between both eyes regarding the grade of papilledema. However, asymmetry in the papilledema grading in IIH was not an uncommon finding. Several mechanisms have been suggested to explain such asymmetry. The concept of compartmentation of the peri-optic subarachnoid spaces in which the peri-optic subarachnoid spaces are partially separated from the suprasellar cisternal spaces, appears to be a contributing factor for the presence of asymmetric papilledema [27, 28]. Other factors, such as decreased elasticity of lamina cribrosa or optic nerve sheet anomaly, have also been suggested to explain asymmetric papilledema [29].

The main limitation of our study is the small sample size. Also, visual acuity was not assessed in relation to electrophysiological measures. In addition, prospective evaluation of these observed electrophysiological parameters over the clinical course of IIH and linking this to visual outcomes would be potentially informative.

## Conclusion

In chronic IIH patients, in addition to the optic nerve dysfunction supported by delayed P100 of VEP, the mfERG revealed a diffuse reduction of P1 amplitude in retinal rings and quadrants and delayed peak time of P1 only in rings 1&2, indicating central retinal changes.

## Data Availability

Authors report that the datasets used and/or analyzed during the current study are available from the corresponding author on reasonable request.
